# Avidity and variable domain spacing strongly influence the therapeutic potency of bispecific antibodies against Crimean-Congo hemorrhagic fever virus

**DOI:** 10.1128/mbio.03202-24

**Published:** 2025-04-16

**Authors:** Albert Wang, Stephanie R. Monticelli, Ariel S. Wirchnianski, Dafna M. Abelson, Ana I. Kuehne, Russell R. Bakken, Marissa Middlecamp, Michael Weingart, Olivia Vergnolle, Zachary A. Bornholdt, Crystal L. Moyer, Jacob L. Berrigan, Brandyn R. West, J. Maximilian Fels, Larry Zeitlin, Andrew S. Herbert, Kartik Chandran, Chowdhury Raihan Bikash, Jonathan R. Lai

**Affiliations:** 1 Department of Microbiology and Immunology, Albert Einstein College of Medicine, Bronx, New York, USA; 2 Virology Division, U.S. Army Medical Research Institute of Infectious Diseases, Fort Detrick, Maryland, USA; 3 Geneva Foundation, Tacoma, Washington, USA; 4 Mapp Biopharmaceutical, Inc., San Diego, California, USA; 5 Department of Biochemistry, Albert Einstein College of Medicine, Bronx, New York, USA; Dartmouth College, Hanover, New Hampshire, USA

**Keywords:** Crimean-Congo hemorrhagic fever virus, monoclonal antibody, bispecific antibody, immunotherapy

## Abstract

Crimean-Congo hemorrhagic fever virus (CCHFV) is an enveloped, negative-sense RNA virus that is spread by ticks across Europe, Africa, and Asia and causes a lethal disease in humans (~30%–40% case fatality). There are currently no approved vaccines or therapeutics. Antibody-based therapeutics targeting the CCHFV surface glycoproteins Gn and Gc, which are responsible for viral attachment and fusion during entry, are a promising therapeutic approach. We previously isolated three broadly neutralizing Gc-targeting human monoclonal antibodies (mAbs) and showed certain cocktails of these mAbs demonstrated synergistic virus neutralization. Furthermore, physical linkage of two of these mAbs into a dual variable domain (DVD) bispecific antibody (bsAb) DVD-121-801 resulted in improved neutralization and therapeutic protection against a lethal CCHFV challenge in mice. However, the molecular requirements for the activity of DVD-121-801, and why it is augmented over monospecific parental mAbs, remain the topic of investigation. Here, we generated a new panel of bsAb variants of DVD-121-801 to explore the spacing and avidity requirements and further optimize its protective efficacy against divergent CCHFV isolates. We evaluated these variants for neutralization, fusion inhibition, and protection with virus-like particles, authentic viruses, and *in vivo* challenge studies. We found that neutralization potency was relatively unaffected by spacing or identity of variable domains within the bsAb, but that one next-generation design employing longer and more flexible linkers between variable domains (DVD-121-801^GS^) had a greater breadth of therapeutic protection. Our efforts highlight the importance of antibody avidity and lead to an improved bsAb variant of DVD-121-801 for further therapeutic development.

**IMPORTANCE:**

Crimean-Congo hemorrhagic fever virus (CCHFV) is a tick-borne virus endemic to Europe, Africa, and Asia that causes severe disease in humans (30%–40% case fatality). There are currently no approved vaccines or therapeutics. In prior work, physical linkage of two Gc-specific monoclonal antibodies (mAbs) targeting distinct epitopes into a dual variable domain (DVD) bispecific antibody (bsAb), termed DVD-121-801, resulted in potent neutralization *in vitro* and therapeutic protection *in vivo*. Here, a panel of variants of this bsAb was developed and evaluated for neutralization potency, fusion inhibition, and therapeutic efficacy, and our work shows this panel to be effective against multiple isolates of CCHFV. Furthermore, incorporating longer, more flexible linkers between variable domains resulted in a lead candidate with improved activity and therapeutic potential compared to the parental bsAb. Utilizing this panel, we also explored the contribution of antibody avidity in antibody-mediated protection against CCHFV infection.

## INTRODUCTION

Crimean-Congo hemorrhagic fever virus (CCHFV) is a widely distributed tick-borne virus that is endemic throughout Europe, Africa, and Asia (1–3). Human infection, most often through tick bites or close exposure to infected livestock, results in non-specific febrile illness that can progress to severe hemorrhagic disease with case fatality rates of 30%–40% (4–6). Although numerous vaccine candidates are in preclinical development, none have been approved by the Federal Drug Administration or European Medicines Agency, and no specific therapies are available to treat CCHFV infections post-exposure (6–8).

CCHFV belongs to the family *Nairoviridae* of enveloped negative-strand RNA viruses in the greater order *Hareavirales*. Its genome comprises three separate segments—small, medium, and large—that, respectively, encode the nucleoprotein (NP) and a non-structural protein (NSs), the glycoprotein precursor complex (GPC) polyprotein, and the RNA-dependent RNA polymerase. GPC is proteolytically processed in host cells to yield multiple glycoproteins—GP160/85, the mucin-like domain, NSm, GP38, Gn, and Gc (6, 9). Recent work has demonstrated that GP38, Gn, and Gc comprise the pre-fusion glycoprotein complex that is incorporated into virions and that mediates viral entry into cells (10, 11). GP38 is also released from infected cells as a separate, secreted polypeptide (12). Elucidation of the three-dimensional structure of Gc in both its putative pre- and post-fusion conformations indicates that it is a Class II fusion protein highly homologous to the E, E2, and Gc proteins of flaviviruses, alphaviruses, and other bunyaviruses, respectively (10, 11, 13–15). Like other Class II fusion proteins, Gc is organized into three domains and adopts a characteristic “trimer of hairpins” structure in its post-fusion conformation (13, 15, 16). In this conformation, domains I and II form a rod-like structure that interact along their entire length to form a trimeric core, and domain III—connected to the domain I–II rods by a linker—forms the outer arm of the hairpin. The “fusion loops” that form a hydrophobic membrane-interacting surface required for viral membrane fusion are located at the distal tip of domain II (15). Recently, Gc was shown to bind the candidate CCHFV entry receptor, a low-density lipoprotein receptor (17–19). Given its central role in CCHFV entry and high sequence conservation among strains (>89%) (15, 20), Gc thus represents an attractive target for therapeutic development.

Multiple clinical studies have shown that the presence of CCHFV-targeting antibodies is correlated with reduced mortality (5). Neutralizing and non-neutralizing monoclonal antibodies (mAbs) targeting Gc and GP38, respectively, have been shown to be protective in animal models of infection (21–23). We previously isolated and identified a panel of Gc-specific mAbs from human CCHFV convalescent donors in Uganda (20). Epitope mapping and structural characterization revealed that these mAbs recognized six distinct antigenic sites in Gc, with mAbs targeting the fusion loop (site 1) and the domain II trimerization interface (site 3) exhibiting broad and high neutralizing potency. Two such mAbs, ADI-37801 and ADI-36121, were among the most potent and targeted conserved residues in sites 1 and 3, respectively (15, 20). On their own, both mAbs were potent neutralizers, but combining ADI-37801 and ADI-36121 in a cocktail resulted in enhanced, synergistic neutralization (20). Furthermore, physically linking the variable fragments (Fvs) of ADI-36121 with ADI-37801 into a dual variable domain (DVD) bispecific antibody (bsAb), termed DVD-121-801, yielded a nearly 10-fold improvement in neutralization potency over the cocktail. In a lethal CCHFV-IbAr10200 mouse model, ADI-36121 and ADI-37801 (alone or cocktail-combined) provided prophylactic protection, but only the bispecific molecule DVD-121-801 could protect mice (80% survival) when administered therapeutically 1 day post-challenge (20). However, the molecular basis for the improved activity of the DVD-121-801 bsAb relative to the cocktail was not explored. Subsequent structural analysis revealed that the two mAbs may act in concert to block membrane fusion (15). Herein, we sought to uncover the requirements for potent neutralization and protection by DVD-121-801, and whether it could be improved by additional bsAb engineering. We generated a panel of related bsAbs that varied in the combinations of epitopes targeted, binding avidity, and distance between Fvs and evaluated them for *in vitro* activity and therapeutic potential in a murine model of lethal challenge with divergent CCHFV isolates *in vivo*. Our results provide new insights into bsAb design for CCHFV and related viruses and afford a second-generation bsAb candidate with enhanced therapeutic potential for further development and evaluation.

## RESULTS

### Bispecific antibody design

The V_H_ domain of ADI-37801 was found to contain several somatic mutations in framework regions relative to the nearest germline progenitor (IGHV4-30-4*09), including an unpaired cysteine at position 49 that may lead to undesired non-disulfide bonds and result in aggregation or diminished activity (24). To remove this potential liability, we generated a new version of the ADI-37801 V_H_ in which Cys49 was reverted to germline (C49W) and incorporated into the parent DVD-121-801. Furthermore, Ser60 in the framework region 3 was also reverted to germline (S60N) in an attempt to further stabilize folding. Neutralization by the reverted DVD-121-801 bsAb was unaffected by these modifications, as described below. For clarity, the mAbs or bsAbs containing the “original” V_H_ including the unpaired Cys49 are hereafter denoted with a “C49” superscript.

**Fig 1:**
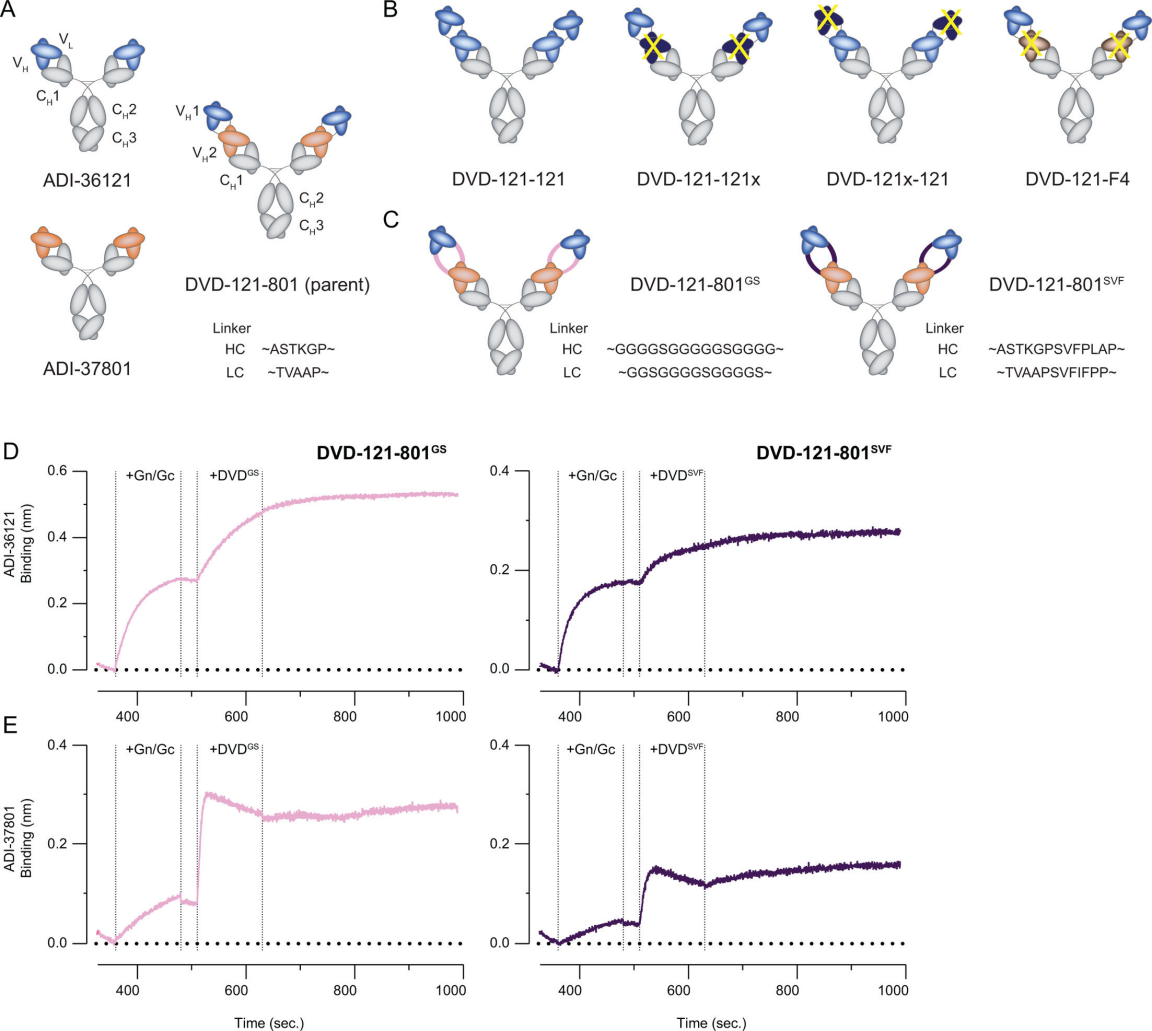
Design of DVD bispecific antibodies. (**A**) Parental molecules. The variable fragments (Fvs) of mAbs ADI-36121 and ADI-37801 are linked into a bispecific molecule in a DVD immunoglobulin format (DVD-Ig), where the Fv of ADI-36121 (“121”) is exterior to that of ADI-37801 (“801”). (**B**) 121-only variants. Binding-impaired variants have one set of Fvs that cannot bind CCHFV-Gc (yellow “X”) either through sequence mutation (dark blue) or substitution with an irrelevant Fv (tan). (**C**) Linker variants. The heavy chain (HC) and light chain (LC) between the two sets of Fvs were extended by the indicated peptide sequence. (**D and E**) Two-phase binding experiments to examine the functionality of Fvs in linker variants. Anti-human probes loaded with ADI-36121 (**D**) or ADI-37801 (**E**) were sequentially associated with recombinant Gn/Gc followed by the indicated DVD-Ig.

In the DVD-Ig format, the C-terminal end of the variable fragment (Fv) of one mAb is linked to the N-terminus of the Fv from a second mAb (25). Between the “inner” and “outer” Fvs are short human-derived linker sequences (~ASTKGP—for heavy chain, and ~TVAAP—for light chain). The parent DVD-Ig, DVD-121-801, contains Fvs from human CCHFV mAbs ADI-36121 (“121”) and ADI-37801 (“801”) as the outer and inner domains, respectively (Fig. 1A).

We previously generated an alternative Gc-targeting DVD-Ig bsAb, DVD-145-801^C49^, that was as neutralizing as DVD-121-801^C49^ but could not protect mice against lethal challenge (20). The “inner” Fvs of DVD-145-801^C49^ were still derived from ADI-37801, but the “outer” Fvs (from ADI-36145) targeted domain III of Gc (15). The differences in DVD-Ig-mediated protection suggested that some component of the ADI-36121 Fv is required, and perhaps sufficient, for efficacy. To explore this hypothesis, we engineered a set of DVD-Ig “121-only variants” (Fig. 1B). We first generated a tetravalent DVD-Ig containing tandem ADI-36121 Fv repeats (DVD-121-121). Based on this construct, variants DVD-121-121x and DVD-121x-121 containing a mutation in the variable light chain (Y32R) that ablated “121” activity in either the “inner” or “outer” domains, respectively, were also generated to test the requirement for one or both domains. Similarly, we replaced the interior set of Fvs with a completely unrelated set of Fvs (from Sudan virus mAb “F4”) that are known to express well (DVD-121-F4) (26).

We next sought to determine if the spacing of the Fvs for DVD-121-801 could influence neutralization potency *in vitro* and protective efficacy *in vivo*. Previous reports have shown that longer linkers between Fvs could provide flexibility to the molecule and improve binding to the inner Fv (27). We thus designed two new “linker variants” of DVD-121-801 (Fig. 1C). In the first variant, DVD-121-801**^SVF^**, the original linkers were extended by seven-residue human-derived sequences reported in Jakob et al. (27): ~ASTKGP**SVFPLAP**—for heavy and ~TVAAP**SVFIFPP**—for light. In the second variant, DVD-121-801**^GS^**, the spacing and flexibility between the two Fvs were extended further by replacement with **G**lycine-**S**erine linkers (15 residues for heavy, and 13 residues for light). A summary of how the Fvs of each DVD-Ig are linked can be found in Table S1. We carried out two-phase biolayer interferometry experiments to test the binding functionality of both sets of Fvs in DVD-121-801^GS^ (Fig. 1D) and DVD-121-801^SVF^ (Fig. 1E). For both linker variants, recombinant Gn/Gc (rGn/Gc) was first bound to the biosensor via ADI-36121 or ADI-37801 prior to incubation with either DVD-Ig. If either set of Fvs in the DVD-Igs were non-functional, a second phase of binding would not be observed as all of the “121” and “801” sites on rGn/Gc would be occupied during the capture phase. Binding to rGn/Gc by both DVD-Igs was observed, providing evidence that the new linkers did not disrupt antigen binding.

### Neutralization of tecVLPs and authentic CCHFV by bsAbs

**Fig 2:**
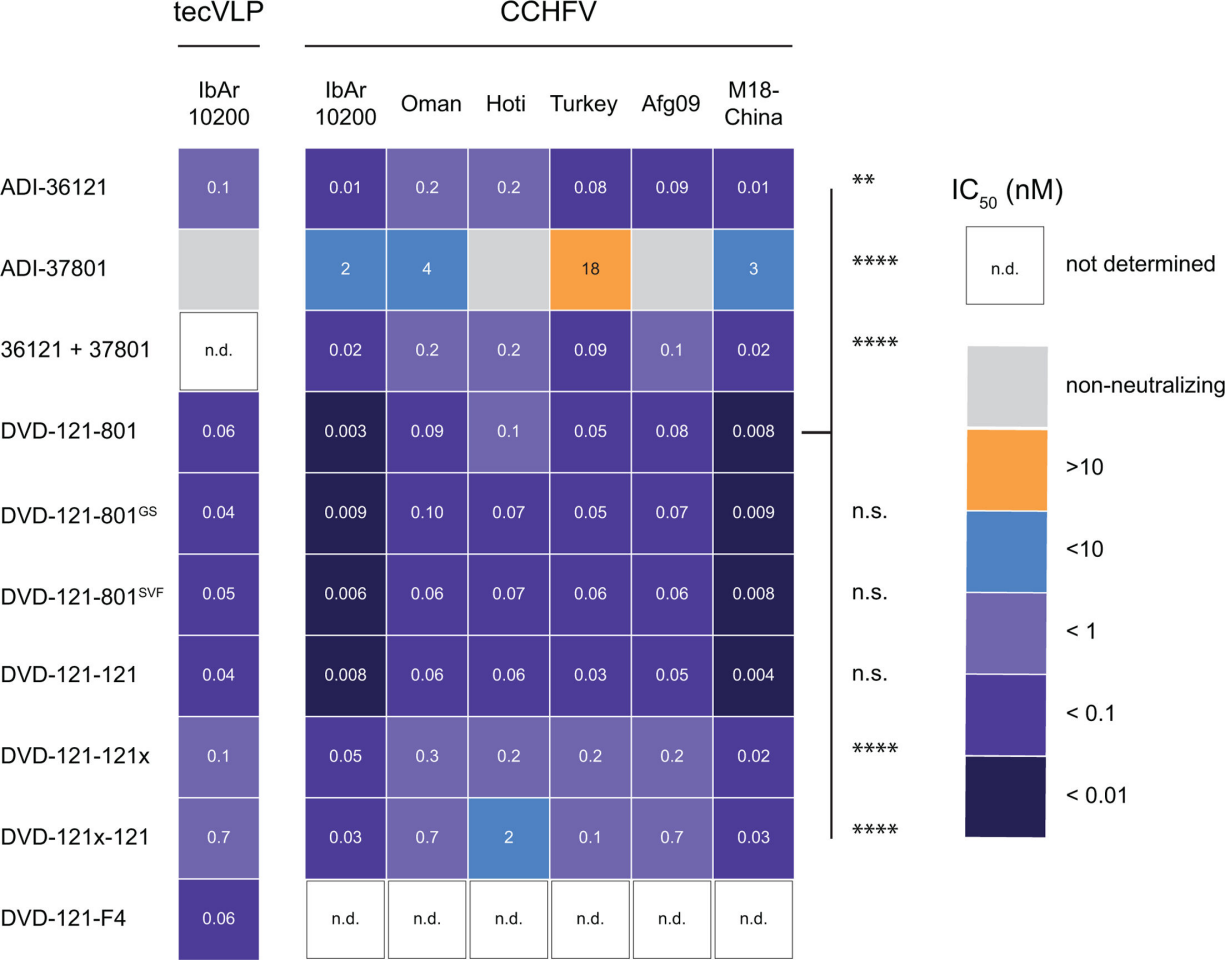
Neutralization profile of the DVD-Ig panel. Heatmap indicating fitted IC_50_ values (values rounded to one significant digit) for each antibody evaluated against tecVLPs (*N* = 3–10, *n* = 9–30) and six authentic strains of CCHFV (*N* = 3–5, *n* = 8–15). IC_50_ values for each antibody were log_10_-transformed and compared by two-way analysis of variance (ANOVA) followed by Šídák’s multiple comparisons tests. Only the comparisons against DVD-121-801 are shown. ****P ≤ 0.0001; ***P ≤ 0.001; **P ≤ 0.01; n.s. *P* > 0.05.

We examined the capacity of bsAbs in our panel to neutralize cell entry by transcription- and entry-competent virus-like particles (tecVLPs) bearing CCHFV IbAr10200 GPC (Fig. 2; Fig. S1A through C). Most of the bsAbs displayed similar neutralization profiles. Incorporation of the SVF and GS linkers neither improved nor diminished activity relative to DVD-121-801, suggesting that spacing between the Fv domains does not have a strong influence on neutralizing activity. However, DVD-121-121x and DVD-121x-121 exhibited reduced neutralization potency; their respective IC_50_ values were 2.5- and 10-fold higher than the molecules DVD-121-801 and DVD-121-121, indicating that the valency of the bsAb correlates to neutralization potency. Interestingly, DVD-121-F4 exhibited an IC_50_ value similar to that of the parents, suggesting that the outer Fv contributes more to neutralization than the inner Fv. The “original” ADI-37801^C49^ had significantly stronger neutralization potency than the germline reverted variant (ADI-37801), but there were no significant differences in neutralization between DVD-121-801^C49^ and DVD-121-801 (Fig. S1D and E). Both DVD-121-801 and DVD-121-801^C49^ were significantly more potent neutralizers than ADI-36121, in agreement with our previous findings (20). Despite the differences, all the bsAbs assayed against tecVLPs reported IC_50_ values less than 1 nM, and thus all constructs had potent neutralizing activity (Table S2).

We next probed the DVD-Igs for neutralization against authentic CCHFV-IbAr10200 and other clinically relevant, diverse isolates of CCHFV spanning multiple clades, including Oman, Hoti, Turkey, Afg09, and M18-China. The same general trends were observed across the isolates (Fig. 2; Fig. S2A through F and S3A through C). DVD-121-801 exhibited potent neutralization (IC_50_ < 1 nM) for all isolates tested (Table S2), significantly better than that of ADI-36121 or ADI-37801, alone or in combination. ADI-37801 was a weaker neutralizing antibody than ADI-36121, failed to neutralize CCHFV-Hoti, and only weakly neutralized CCHFV-Afg09 at high concentrations (Fig. S2C, parental). Both linker variants DVD-121-801^GS^ and DVD-121-801^SVF^ were similar to the parental DVD-121-801 in neutralizing all CCHFV isolates (Fig. 2; Fig. S2A through F and S3A through C). Interestingly, DVD-121-121 also neutralized all isolates as well as the parent DVD-Ig, suggesting that the “801” Fv may not be required. In contrast, the 121-only variants that lacked functional Fvs in both domains (DVD-121-121x, DVD-121x-121) showed a significant reduction of neutralization IC_50_ across all isolates as compared to both DVD-121-801 (~2- to 20-fold) and DVD-121-121 (~3- to 33-fold), suggesting that avidity contributes significantly to neutralizing potency. DVD-121x-121 (with a non-functional outer domain Fv pair) was less neutralizing than DVD-121-121x against CCHFV-Hoti (Fig. S2C, 121-only variants). However, this trend was not observed for the other authentic strains. Since DVD-121-F4 was functionally similar to DVD-121-121x, it was not further examined. Comparing across isolates, all the tested antibodies generally neutralized the IbAr10200 and M18-China isolates of CCHFV at an order of magnitude greater than the other isolates tested or against tecVLPs (Fig. 2).

### Fusion inhibition of tecVLPs by bsAbs

**Fig 3:**
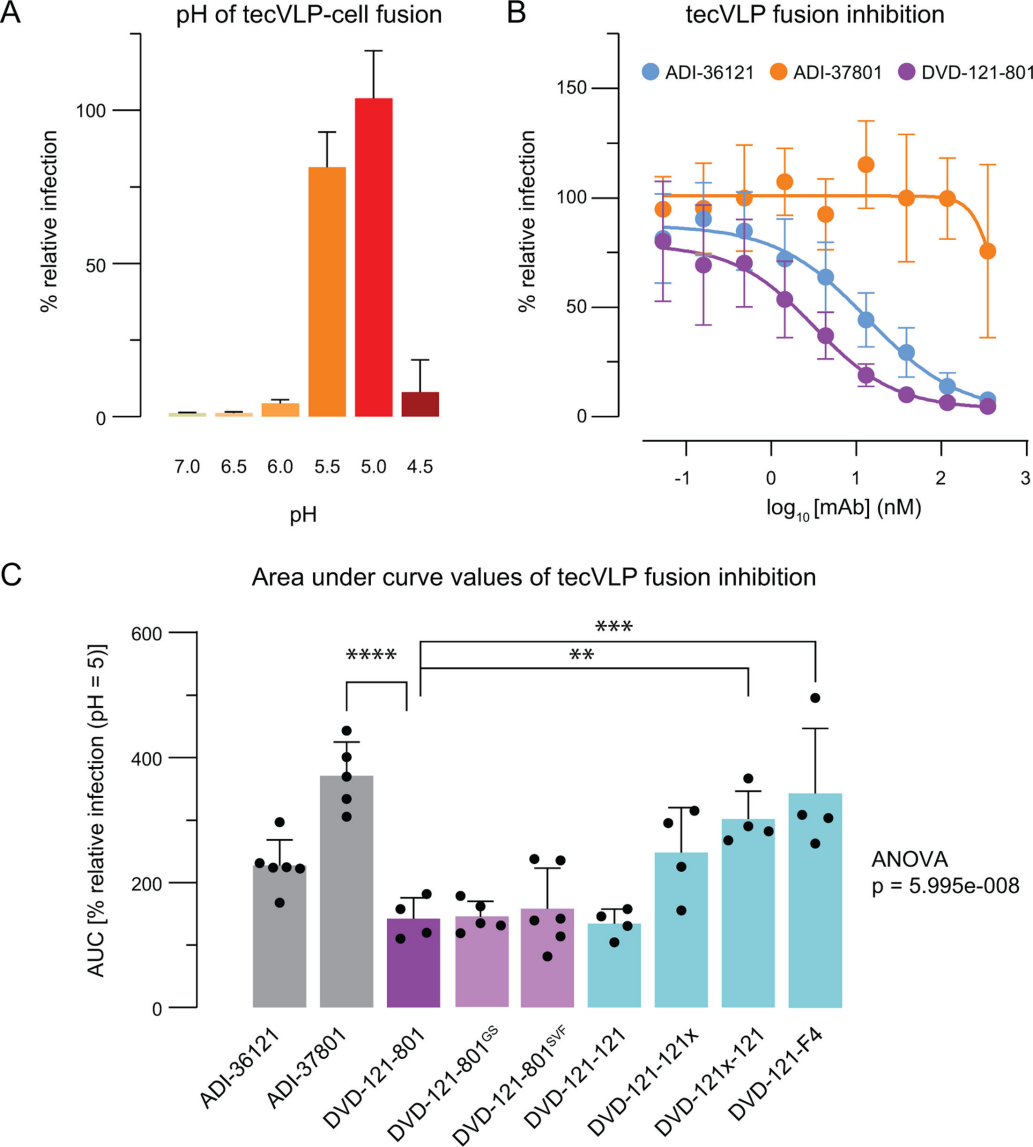
Fusion inhibition profile of DVD-Ig panel. (**A**) Fusion and resulting infection of Vero cells by tecVLPs (Oman) at different pH conditions (*N* = 2, *n* = 6). (**B**) Fusion inhibition plots of parental mAbs and bsAb DVD-121-801 (*N* = 4–6, *n* = 8–18). (**C**) AUC values were calculated from antibody fusion inhibition data. The greater the AUC value, the lower the inhibition. AUC values for each antibody were compared by one-way ANOVA. Subsequent Šídák’s multiple comparisons test compared all AUC values against the parent bsAb, DVD-121-801. Non-significant comparisons are not shown. ****P ≤ 0.0001; ***P ≤ 0.001; **P ≤ 0.01.

In previous work, ADI-36121 and ADI-37801 were shown to, respectively, bind CCHFV Gc at the domain II base and domain II fusion loops (15). The authors hypothesized that ADI-36121 binding disrupts the higher-order spike lattice in virions, thereby increasing the accessibility of the fusion loops to ADI-37801 and contributing to synergistic neutralization by DVD-121-801. To evaluate the hypothesis that DVD-121-801 and related bsAbs neutralize the virus via fusion inhibition, we developed a novel fusion assay using tecVLPs bearing CCHFV-Oman GPC. TecVLPs were allowed to attach to the surface of target cells at 4°C, and cells were subsequently exposed to warmed media titrated to different acidic pH values (pH 4.5–7.0) to trigger fusion and infection at the cell surface. The lysosomotropic agent NH_4_Cl was included in the media to neutralize the endo/lysosomal compartment and inhibit endosomal acid-triggered viral membrane fusion. Exposure of tecVLP-bound cells to media titrated to pH 5.0–5.5 was necessary for viral entry, concordant with viral fusion from or near the plasma membrane and a threshold of pH ~5.5 for viral fusion triggering (15, 28). Further reduction of extracellular pH to 4.5 dramatically reduced viral entry (Fig. 3A). Accordingly, we evaluated the DVD-Ig panel for fusion inhibition at pH 5.0. We observed that ADI-36121 inhibited fusion (Fig. 3B). DVD-121-801 inhibited fusion at lower concentrations with respect to ADI-36121, although this difference by area under the curve (AUC) was not statistically significant (Fig. 3C; Fig. S4C and D). Surprisingly, ADI-37801 did not inhibit fusion, even at mAb concentrations of 350 nM. Similar to our neutralization results, DVD-Igs with two functional sets of Fvs (DVD-121-801^GS^, DVD-121-801^SVF^, DVD-121-121) displayed no significant differences in their AUC values to inhibit fusion as compared to the parent bsAb (Fig. 3C; Fig. S4A, C, and D). DVD-Igs containing a non-functional set of Fvs (DVD-121-121x, DVD-121x-121, DVD-121-F4) showed comparable or worse fusion inhibition to that of ADI-36121 or the parent bsAb, suggesting that functional Fvs in both the inner and outer domain are required for optimal inhibition of viral membrane fusion (Fig. 3C; Fig. S4B through D).

### DVD-121-801^GS^ treatment confers partial protection against multiple CCHFV isolates

**Fig 4:**
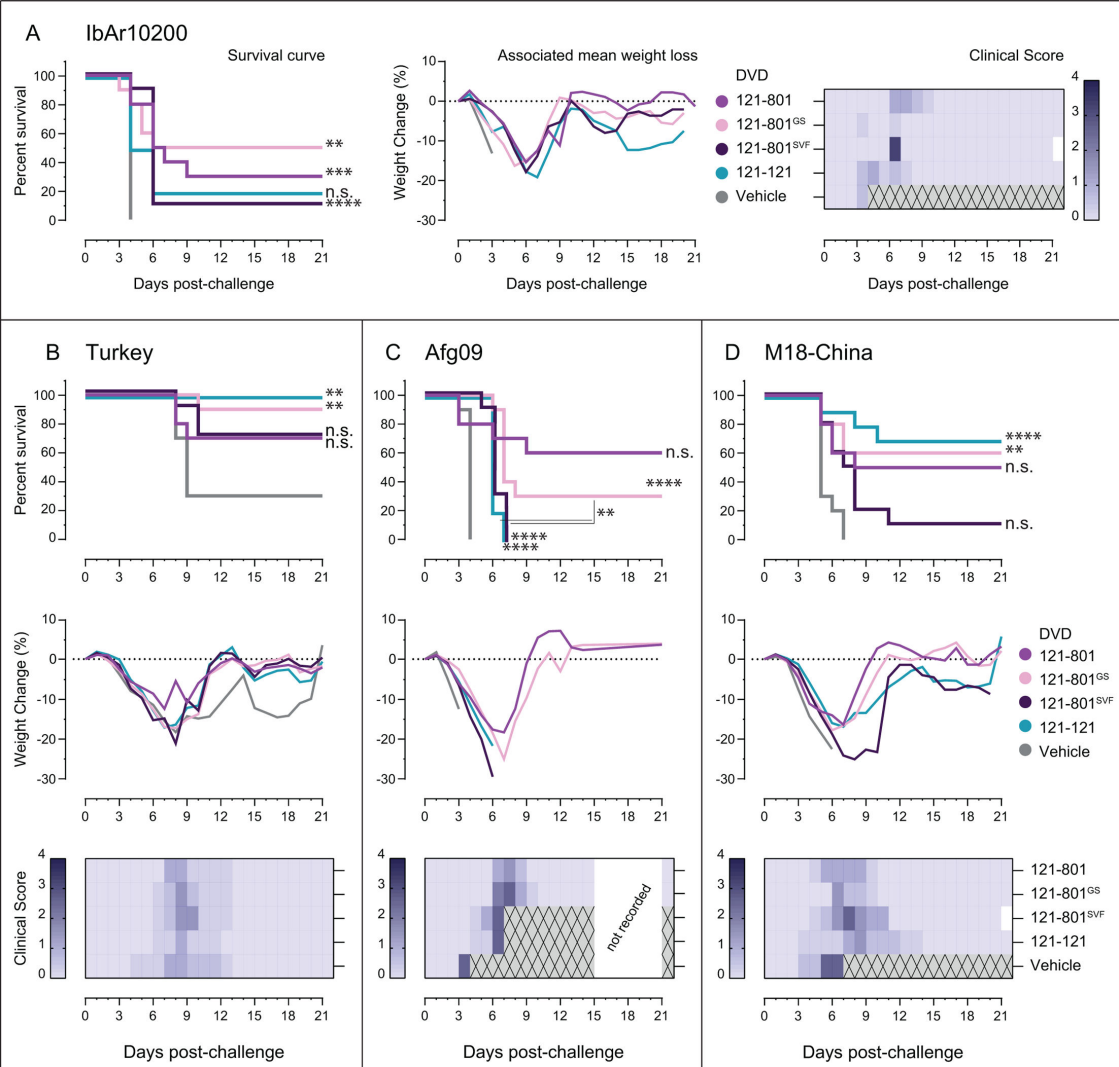
DVD-121-801^GS^ affords partial broad therapeutic protection. (**A**) Type 1 Interferon α/β receptor knockout (IFNAR1^−/−^) mice were exposed intraperitoneally (IP) to 100 PFU of CCHFV-IbAr10200 and treated IP with 1 mg of each bsAb (*N* = 2, *n* = 5) 1 day post-challenge. Survival curve and associated mean weight loss and clinical score are shown. (**B–D**) Other groups were exposed to 1,000 PFU of CCHFV-Turkey (**B**) or to 100 PFU of CCHFV-Afg09 or -M18-China (**C and D**) and treated with bsAb similarly. Associated mean weight loss and clinical score are shown below each survival plot. Survival curves for each challenge strain were compared between treatment groups and vehicles by Mantel-Cox tests (Bonferroni-corrected α = 0.005). Additionally, the DVD-121-801^GS^ treatment group was compared statistically to DVD-121-801^SVF^ and DVD-121-121 in the CCHFV-Afg09 group. The study period encompassed 21 days after the initial challenge. White boxes indicate no data recorded while gray boxes with an "X" indicate all mice in that group had succumbed to disease. ****P ≤ 0.0001; ***P ≤ 0.001; **P ≤ 0.01; n.s. *P* > 0.05.

We next evaluated our bsAb panel for therapeutic efficacy in a lethal mouse model of CCHFV-IbAr10200 infection (29, 30). We began by evaluating the most potent neutralizing bsAbs: the GS and SVF linker variants, as well as DVD-121-121. Type 1 interferon α/β receptor knockout (IFNAR1^−/−^) mice were challenged with 100 particle forming units (PFU) of CCHFV-IbAr10200 and treated with 1 mg/mouse of bsAb 1 day post-challenge. All vehicle-treated animals succumbed to infection 4 days post-challenge, and overall, treatment with any of the bsAbs significantly improved survival, with the exception of DVD-121-121 (Fig. 4A). Mice receiving DVD-121-801^GS^ had the highest level of protection with 50% survival. Treatment with this bsAb also resulted in less severe clinical scores throughout the course of disease, with DVD-121-801 or DVD-121-121-treated groups experiencing a more prolonged disease course as measured by weight loss and clinical score (Fig. 4A).

We expanded our panel for evaluation against multiple clinical isolates of CCHFV, including Turkey, Afg09, and M18-China. Against CCHFV-Turkey, vehicle-treated mice lost weight and became sick 3–8 days post-challenge, and 70% of mice succumbed by 9 days post-challenge (30% survival; Fig. 4B). All vehicle-treated animals challenged with CCHFV-Afg09 and CCHFV-M18-China succumbed to infection 4 to 7 days post-challenge (Fig. 4C and D). Treatment by the parent molecule DVD-121-801 afforded protection against all three isolates with 50%–70% survival, although against CCHFV-Turkey the level of survival was not statistically distinguishable from vehicle control (Fig. 4B through D). DVD-121-121 significantly improved mice survival against CCHFV-Turkey (100%; Fig. 4B) and CCHFV-M18-China (70%; Fig. 4D). Although none of the DVD-121-121-treated mice survived the CCHFV-Afg09 challenge, there was a significant delay in time to death (6–7 days vs 3 days). Treatment with DVD-121-801^SVF^ did not significantly improve survival relative to control against CCHFV-Turkey (Fig. 4B) but resulted in a delay in morbidity against CCHFV-Afg09 and M18-China (Fig. 4C and D). Despite these improvements in survival, DVD-121-801^SVF^ treated mice experienced a more prolonged disease course evidenced by weight loss and clinical scores of disease. DVD-121-801^GS^-treatment reduced weight loss and clinical signs of disease and resulted in significant mice survival against lethal disease following challenge with each isolate of CCHFV tested: CCHFV-Turkey—90%, CCHFV-Afg09—30%, CCHFV-M18-China—60% (Fig. 4B through D). Together with protection data for CCHFV-IbAr10200—50% survival, DVD-121-801^GS^ treatment significantly improved survival in mice against a diverse range of CCHFV isolates relative to other bsAbs in the panel.

### DVD-121-801^GS^ is stable for long-term storage

Finally, to explore the developability of the DVD-Ig molecules, we examined the stability of parent DVD-121-801 as well as DVD-121-801^GS^ upon storage for up to 60 days at 4°C or at 40°C (Fig. S5). When stored at 4°C, there was minimal degradation of either bsAb as determined by size exclusion chromatography. When stored at 40°C, there was some degradation of bsAbs, but >85% of the samples were monomeric even after prolonged heating. These results highlight the stability of both molecules and suggest favorable developability properties.

### BsAb-mediated protection requires avidity

**Fig 5:**
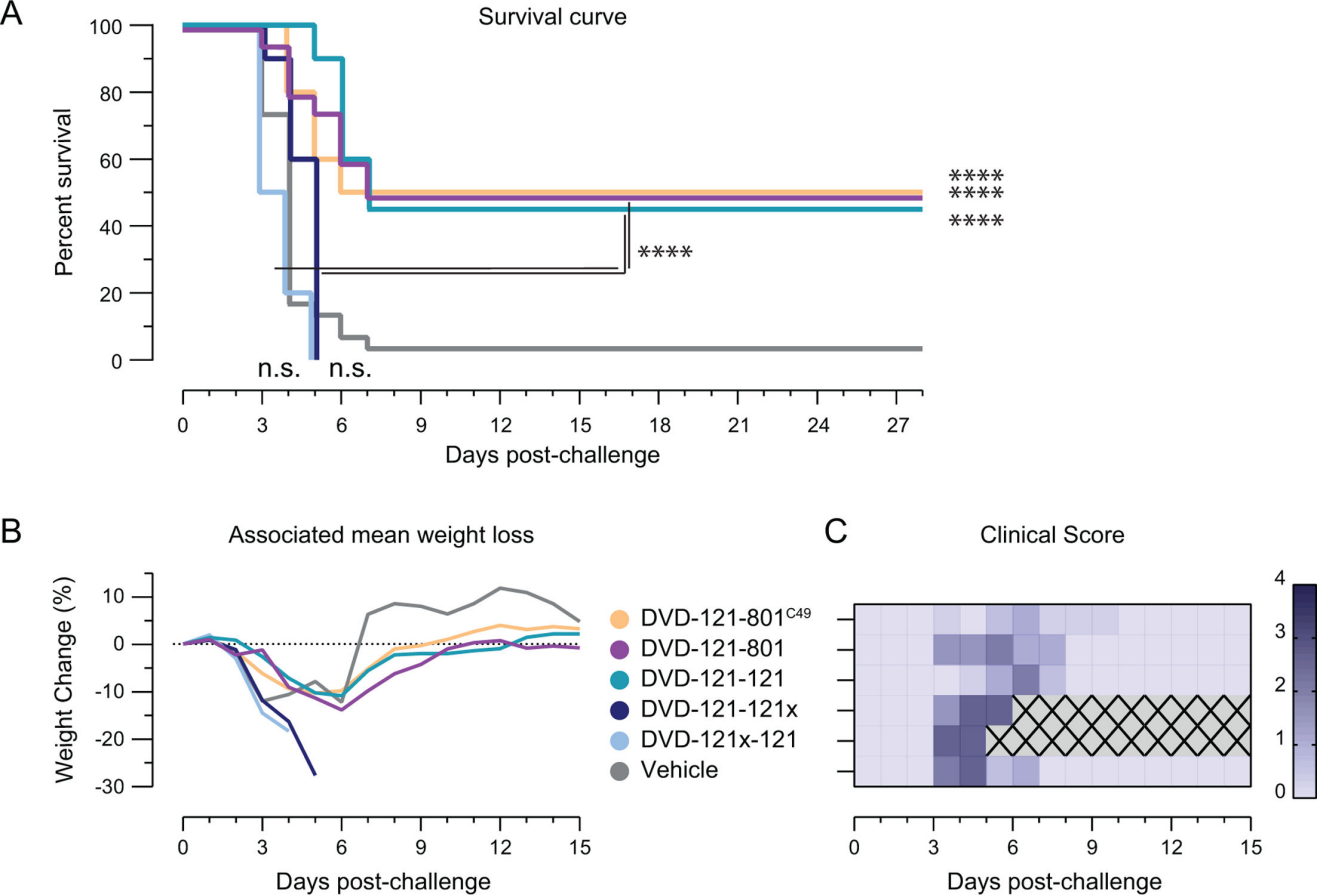
Avidity drives bispecific antibody-mediated protection against CCHFV IFNAR1^−/−^ mice (*n* = 10–20) were challenged with 100 PFU of CCHFV-IbAr10200 and treated with 1 mg of each indicated bispecific antibody or vehicle 1 day post-challenge. (**A**) Survival outcomes were monitored for 28 days post-challenge and compared against vehicle control by Mantel-Cox tests (Bonferroni-corrected α = 0.00417). Additionally, the survival of DVD-121-801 and DVD-121-121 were compared with DVD-A121-121x and DVD-121x-121. Associated weight change (**B**) and clinical score (**C**) were monitored until 15 days post-challenge. Gray boxes with an “X” indicate all mice in that group had succumbed to disease.

Given that DVD-121-121x and DVD-121x-121 were potently neutralizing despite each having only a single set of functional Fv domains, we next tested them for protective efficacy against CCHFV-IbAr10200 in comparison to DVD-121-121. We included both DVD-121-801^C49^ and the germline-reverted version of DVD-121-801 in our study (20). As before, the majority of vehicle-treated mice succumbed to lethal infection by 4 days post-challenge (90%; Fig. 5A). Both versions of the parent bsAbs significantly improved survival, with 50% of the treated mice surviving the study period. DVD-121-121 achieved the same level of protection (50%) as the parent molecules and showed similar protection against weight loss (Fig. 5B). Strikingly, all the mice treated with bsAbs DVD-121-121x or DVD-121x-121 rapidly lost weight, registered high clinical scores of disease, and died by day 5 post-challenge (Fig. 5A through C). These results highlight the crucial role of avidity in bsAb protection.

## DISCUSSION

Bispecific antibodies offer a promising modality for antiviral therapeutics. DVD-121-801 is a bispecific antibody whose component Fvs target CCHFV Gc at two distinct antigenic sites: the trimerization domain (“121”) and the fusion loops (“801”). Previous sequence alignments and structural analysis of ADI-37801 and ADI-36121 Fabs in complex with CCHFV Gc demonstrated that their epitopes are highly conserved among orthonairoviruses (15). Specifically, 11 of the 12 residues that constitute the ADI-37801 epitope were conserved among all 15 CCHFV isolates examined, with one residue (position 1,193) conserved for 14 of them (15). Similarly, across 15 CCHFV isolates, 17 of the 22 residues that form the epitope of ADI-36121 were completely conserved. Of the remaining five residues, four residues (position 1,146, 1,227, 1,228, and 1,276) were conserved across 14 isolates (15). Therefore, bsAbs containing Fvs from these two mAbs were predicted to have broad reactivity. Herein, we generated a new panel of bsAbs to investigate the molecular basis of DVD-121-801’s protective activity *in vivo* (20) and to assess the cross-clade therapeutic efficacy of DVD-121-801 and its derivatives.

Previous work isolated three potent, Gc-targeting mAbs: ADI-36121, ADI-37801, and ADI-36145. At that time, structural characterization was incomplete, and four bispecific antibodies (DVD-Ig format) were generated from the three indicated mAbs to take advantage of observations of neutralization synergy (20). The combinations that showed enhanced neutralization were DVD-121-801^C49^ and DVD-145-801^C49^, in which the Fvs of “801^C49^” were the “inner” set of domains relative to the other Fvs. Only DVD-121-801 protected mice against lethal challenge (20). In the Gc trimer, the fusion loops are transiently accessible on virus particles (15), and a recently published pre-fusion-stabilized heterotrimeric CCHFV glycoprotein complex structure (GP38-Gn-Gc) revealed that an N-linked glycan (Asn557) on Gn may mask the viral fusion loops from premature insertion into target membranes and sequester access by neutralizing antibodies (11). As indicated in the Introduction, ADI-37801 targets the fusion loops, thereby inhibiting Gc trimer insertion, while ADI-36121, which targets the domain II trimerization interface, binds laterally to the domain II base; this binding epitope is completely buried and inaccessible in the post-fusion trimer of Gc (15). Mishra and Hellert et al. (15) demonstrated that the affinity of ADI-36121 binding to the Gc monomer was 200× higher than that to the Gc trimer and suggested that the “121” Fv of DVD-121-801 disrupts Gc breathing dynamics to allow the “801” Fv to bind to the fusion loops (15).

We lengthened the linker between the “121” and “801” Fvs to try to improve access to the interior “801” Fv, thereby generating DVD-121-801^SVF^ and DVD-121-801^GS^. Both linker variants were indistinguishable from the parent DVD-121-801 in terms of neutralization and fusion inhibition. *In vivo*, treatment with the parent DVD-Ig resulted in 50%–60% survival in mice against the clinical isolates of CCHFV. Of the two linker variants, the GS linker variant significantly protected mice against challenge by multiple isolates, while the SVF linker variant was largely ineffective. DVD-121-801^GS^ may be a more effective molecule due in part to its glycine-serine linker flexibility (31). This flexibility may allow DVD-121-801^GS^ to not only contort in such a way that both Fvs bind effectively, but also overcome potential CCHFV isolate differences in surface lattice presentation or breathing dynamics. On the other hand, the DVD-121-801^SVF^ has bulky side chains in its linkers with a collective six proline and three phenylalanine residues in each arm that could impede synergistic binding.

The 121-only bsAb variants were designed to examine the role of avidity and the positional role of the “121” Fv. DVD-121-121 was a more potent bsAb both *in vitro* and *in vivo* compared to binding-impaired 121-only variants. However, the post-exposure efficacy of DVD-121-121 in mice was variable and dependent on the virus isolate, despite the antigenic epitopes targeted by “121” Fv being equivalent across all four isolates. Even so, we demonstrated the importance of avidity in targeting CCHFV Gc. Fels et al. demonstrated that the relative positioning of the “121” and “801” Fvs affects neutralization (20); the reciprocal molecule to DVD-121-801^C49^, DVD-801^C49^-121 (where the “outer” domains were drawn from ADI-37801^C49^) did not improve neutralization over ADI-37801^C49^. We show that by increasing the valency of a molecule with the same Fv, we can overcome the Fv positioning requirements in these bsAbs. Given the hypothesis that “121” transiently disrupts Gc breathing dynamics, the increased “121” valency on DVD-121-121 may allow the bsAb to disrupt the surface glycoprotein lattice more easily.

Viral neutralization potency partially predicted the *in vivo* protective efficacy of the bsAbs panel. DVD-121-801 and its variants with four functional Fvs had authentic neutralization IC_50_ values that were approximately 10-fold lower (i.e., better) than the component mAbs (ADI-36121 or ADI-37801) or DVD-121x-121 and DVD-121-121x for all isolates tested. As expected, the antibodies with the higher (worse) IC_50_ values failed to protect mice in a therapeutic setting. DVD-121-801, its linker variants, and DVD-121-121 were extremely potent and registered neutralization IC_50_ values in the range of 10^−12^–10^−11^ molar. Potentially, serum half-life or Fc effector function could also be contributing to protective mechanisms which may explain differences in neutralization potency and *in vivo* protection.

We have identified a second-generation bsAb, DVD-121-801^GS^, that provides the greatest cross-isolate protective efficacy in our panel and is relatively stable (Fig. S5). While there are some differences in survival percentage across CCHFV isolates, treatment by DVD-121-801^GS^ was significantly protective against all isolates tested. Any isolate-dependent differences in protection could be attributed to variations in general breathing dynamics, but additional structural studies would be required to explore this hypothesis. Given that 100% protection was not achieved by administering the bsAb alone, the treatment could be optimized via further antibody engineering in the Fc region. In conjunction with targeting entry, mAbs that target GP38 could be added in an antibody cocktail to also target pathogenicity (22, 23, 32). Overall, we have generated a new molecule, DVD-121-801^GS^, that improves the activity and antiviral breadth of the novel bsAb DVD-121-801 in highly lethal murine models of CCHFV challenge and that is suitable for evaluation in a large-animal model of CCHFV infection and disease.

## MATERIALS AND METHODS

### Cell culture

Vero and VeroE6 cells, a spontaneously immortalized cell line isolated from the kidney of adult African green monkey kidney cells, were previously purchased from and authenticated by the American Type Culture Collection (CCL-81, RRID: CVCL_0059). Vero/VeroE6 cells were cultured in Dulbecco’s Modified Eagle Medium (DMEM; ThermoFisher Scientific [TFS]) supplemented with 2%/10% heat-inactivated fetal bovine serum (ΔFBS; Bio-Techne/Gemini-Bio), 1% penicillin-streptomycin (P/S; TFS), and 1% GlutaMAX (TFS). BSR-T7 cells (RRID: CVCL_RW96), a spontaneously immortalized cell line isolated from the kidney of a Golden hamster, stably express T7 RNA polymerase and were a kind gift from K.-K. Conzelmann. BSR-T7 cells were cultured in DMEM supplemented with 10% FBS, 1% P/S, and 1% GlutaMAX. These cells were not authenticated following gifting. The above adherent cells were maintained in a humidified 37°C incubator supplied with 5% CO_2_. ExpiCHO-S cells (TFS) were cultured in ExpiCHO Expression Medium (TFS) supplemented with 1% P/S and maintained in a humidified shaking incubator (37°C, 8% CO_2_, 125 rpm).

### Virus stocks

The authentic CCHFV isolates CCHFV-IbAr10200, CCHFV-Afg09-2990 (labeled as “Afg09”), CCHFV-Turkey2004 (labeled as “Turkey”), CCHFV-Oman-1998091666 (labeled as “Oman”), CCHFV-M18-China, and CCHFV-Kosovo Hoti (labeled as “Hoti”) were used in this study.

### Expression and purification of mAbs and bsAbs

pMAZ-IgH and pMAZ-IgL vectors encoding DVD-121-801^C49^, DVD-121-801^GS^, DVD-121-801^SVF^, and DVD-121-F4 were subcloned. The parent DVD-Ig was cloned as previously described (20). The outer variable domains for DVD-121-801^GS^ were linked via ~GGGGSGGGGGSGGGG~ and ~GGSGGGGSGGGGS~ while those for DVD-121-801^SVF^ were linked by ~ASTKGPSVFPLAP~ and ~TVAAPSVFIFPP~ for the heavy and light chains, respectively. DVD-121-F4 contained the canonical DVD-Ig linker (25) and the F4 antibody (26). Sequences were verified by Sanger sequencing. Antibodies were expressed by co-transfecting ExpiCHO cells (TFS) as per the manufacturer’s instructions with pMAZ-IgH and pMAZ-IgL for each respective antibody. Antibodies were purified as previously described (33). Briefly, cells were pelleted, and the supernatant was then incubated and stirred with protein A resin for 2 h at 4°C. Antibodies were purified using the PierceTM Gentle Ag/Ab binding and elution buffers (TFS) following the manufacturer’s protocol. Eluted antibody was buffer exchanged into Hepes buffer (200 mM NaCl, 150 mM HEPES [pH 7.4]) and concentrated using Amicon centrifugal filter units (Millipore Sigma) with a nominal molecular weight cutoff of 50 kDa.

Antibodies were expressed transiently in ExpiCHO cells (TFS) and purified from cell supernatants using a GE MabSelect SuRe LX protein A affinity chromatography column on an AKTA pure fast protein liquid chromatography system. The antibodies were eluted using Pierce IgG elution buffer and neutralized with a 2 M Tris base to a pH of ~7.

### Biolayer interferometry of bsAbs

The antibody binding properties were determined by biolayer interferometry using the Octet Red (Fortebio, Pall LLC). CCHFV IbAr10200 recombinant, biotinylated Gn/Gc was loaded onto streptavidin biosensors subsequently followed by mAb or bsAb association and dissociation as previously described (20).

### Generation of tecVLPs

The amino acid sequences for the IbAr10200 and Oman-199809166 were derived from GenBank M-segment sequences with accession numbers NC_005300 and KR864901, respectively. tecVLPs bearing CCHFV glycoproteins were generated as previously described (20, 34). Briefly, BSR-T7 cells were transfected with five plasmids separately encoding the CCHFV NP, GPC, polymerase (L), T7 polymerase, and a Nano-Glo luciferase minigenome in the absence of P/S. Fifteen hours post-transfection, transfection medium was replaced on cells with P/S-containing DMEM growth medium. Sixty hours post-transfection, tecVLP-containing supernatants were collected, clarified by low-speed centrifugation, and pelleted by ultracentrifugation at 25,000 rpm for 2.5 h. Pelleted tecVLPs were resuspended in DMEM overnight before aliquoting and storage at −80°C.

### TecVLP neutralization

TecVLPs bearing IbAr10200 glycoproteins were titered after generation, and an empirical dilution was chosen for neutralization assays such that the maximum luminescence signal was two orders of magnitude higher than that of the background. Vero cells were seeded in 96-well flat-bottomed, white cell culture plates (Corning) at 18,000 cells per well 24 h before infection. Threefold serial dilutions were performed for each antibody. TecVLPs were then incubated with the antibodies for 1 h at 4°C before the antibody/tecVLP mixtures were added to the cells. Infection was allowed to proceed in a humidified 37°C incubator supplied with 5% CO_2_ for 14–16 h. The infection medium was then dumped, cells were washed once with phosphate-buffered saline (PBS), and a luminescence signal was developed using the Nano-Glo luciferase assay system (Promega) per the manufacturer’s instructions. Infectivity was quantified by luminescence signal using Cytation 5 (v 3.1.2) cell imaging multimode reader (Biotek/Agilent).

### Authentic virus neutralization

Neutralization assays were conducted as described previously, with modifications (20, 23). In brief, CCHFV-IbAr10200, CCHFV-Afg09, CCHFV-Turkey, CCHFV-Oman, CCHFV-M18-China, or CCHFV-Hoti were incubated with serial fourfold dilutions of mAbs (at a starting concentration of 50 nM) for 1 h at 37°C. The antibody virus mixture was added to monolayers of VeroE6 cells in a 96-well plate at a final multiplicity of infection of 0.08 (IbAr10200 or M18-China), 0.2 (Afg09), 0.04 (Turkey), 0.05 (Oman), or 0.03 (Hoti) and incubated for 1 h at 37°C. The infection medium was then removed, and a fresh cell culture medium without mAb was added. After 48 (IbAr10200, Afg09, or M18-China) or 72 (Turkey, Oman, or Hoti) hours post-infection, the culture medium was removed, and plates were submerged in 10% neutral buffered formalin and fixed for at least 24 h at 4°C. Plates were removed from formalin, washed with PBS three times, and permeabilized with 0.2% Triton-X for 10 min at room temperature. Following permeabilization, plates were washed three times with PBS and blocked with blocking buffer (Cell Stain Buffer; TFS) for 2 h at 37°C. Infected cells were detected by successive incubation with CCHFV-specific anti-nucleocapsid antibody 9D5 (3 µg/mL; BEI NR-40270) and secondary detection antibody (goat anti-mouse) conjugated to AlexaFluor 488 (diluted 1:2,000; Invitrogen). Percent infection was determined using a Cytation 5 high-content imaging instrument and data analysis was performed using Gen5.11 software (BioTek).

### Neutralization (tecVLP and authentic virus) analysis

Luminescence or fluorescence values were normalized to the average signal from infected cells that received no antibody. Absolute or relative IC_50_ values for each antibody were derived from fitted inhibition curves using non-linear regression (variable slope, four parameters; GraphPad Prism 10.0.3). Absolute IC_50_ values (Fig. S2D) were compared by Welch analysis of variance (ANOVA) with Dunnett’s T3 multiple comparisons tests on pre-selected comparisons indicated in the corresponding figure legend after qualifying the data for normality and homoscedasticity (Fig. S2E). Relative IC_50_ values were log-transformed to fulfill normality requirements (Fig. S4A and B) and fit the data for two-way ANOVA comparisons (Fig. S4C; Fig. 2A) with Dunnett’s multiple comparisons test.

### Fusion optimization

Vero cell monolayers plated in 96-well flat-bottomed, white cell culture plates (Corning) were cooled on ice for 10 min prior to the replacement of media with a predetermined dilution of tecVLPs bearing Oman-199809166 glycoproteins in Vero media. The virus was spinoculated onto cells at 2,500 rpm for 1 h at 4°C. Cells were kept on ice while unbound virus inoculum was removed and replaced with a panel of fusion media (20 mM NH_4_Cl in DMEM/F-12 50/50 [Corning]) with pHs ranging from 7.0 to 4.5. Fusion was induced upon incubating plates in a 37°C water bath for 5 min. Cells were placed back on ice, and fusion medium was exchanged with Vero medium supplemented with 20 mM NH_4_Cl and then incubated at 37°C 5% CO_2_ for 14–15 h. Media were discarded, and cells were washed with PBS prior to development with NanoGlo Luciferase Assay (Promega) per the manufacturer’s instructions. Infectivity was quantified by luminescence signal using the Cytation 5 cell imaging multimode reader (Biotek/Agilent).

### Fusion inhibition assay

TecVLPs bearing Oman-199809166 glycoproteins were titered after generation, and an empirical dilution was chosen for fusion assays such that the maximum luminescence signal was two orders of magnitude higher than that of the background. Vero cells were seeded in 96-well flat-bottomed, white cell culture plates 24 h before infection to form a monolayer. Threefold serial dilutions were performed for each antibody, and tecVLPs were then incubated with the antibodies for 1 h at ambient temperature. Cells and the antibody/tecVLP mixtures were chilled to 4°C before the antibody/tecVLP mixtures were added onto the cells and spinoculated (~900 g) for 1 h at 4°C. On ice, the infection medium was removed from the cells and substituted with fusion medium (20 mM NH_4_Cl in DMEM/F-12 50/50 [Corning], titrated to pH = 5). Spinoculated cells were transferred to a 37°C water bath for 5 min to allow for fusion before they were placed back onto ice. The fusion medium was removed and replaced with Vero growth media supplemented with 20 mM NH_4_Cl. Cells recovered in a humidified 37°C incubator supplied with 5% CO_2_ for 14–16 h. The medium was then dumped, cells were washed once with PBS, and a luminescence signal was developed using the Nano-Glo luciferase assay system (Promega) per the manufacturer’s instructions. Infectivity was quantified by luminescence signal using Cytation 5 (v 3.1.2) cell imaging multimode reader (Biotek/Agilent).

Luminescence values were adjusted for background and normalized to the average signal from infected cells that received no antibody. AUC values for each antibody were calculated through GraphPad Prism 10.0.3 (baseline *Y* = 0, ignore peaks < 10% [*Y*min, *Y*max]). After qualifying the data for normality and homoscedasticity (Fig. S5D and E), AUC values were compared by ordinary one-way ANOVA with Šídák’s multiple comparisons test on pre-selected comparisons (GraphPad Prism 10.0.3) indicated in the corresponding figure legend.

### Animal challenge

A total of 5–12-week-old male and female B6(Cg)-*Ifnar1^tm1.2Ees^/J* mice (IFNAR^−/−^; The Jackson Laboratory; strain #028288) (30, 35) were exposed intraperitoneally (IP) to either 100 PFU of CCHFV-Ibar10200, CCHFV-Afg09, or CCHFV-M18-China or to 1,000 PFU of CCHFV-Turkey. Mice were treated IP with 1 mg of the indicated bsAb (on average 40 mg/kg per mouse), or an equivalent volume (200 µL) of PBS vehicle 24 h (+1 day) post-challenge. Animals were observed daily for clinical signs of disease and morbidity for 21 days post-challenge. Mice were scored on a 4-point IACUC-approved grading scale: 1 is decreased grooming and/or ruffled fur, 2 is subdued behavior when un-stimulated, 3 is lethargy, hunched posture, and/or subdued behavior even when stimulated, and 4 is bleeding, unresponsiveness, severe weakness, and/or inability to walk. Mice scoring a 4 were considered moribund and euthanized based on IACUC-approved methods. Mice scoring a 3 were observed at a minimum of twice daily.

### Stability assay

Purified antibodies were formulated in 10 mM histidine pH 6, 5% sorbitol, and concentrated to 20 mg/mL, at which time PS80 was added to a final concentration of 0.05%. The antibodies were stored at 4°C or 40°C. At designated intervals, samples were taken for analysis by size exclusion chromatography. Samples were run on a Tosoh TSKgel SuperSW3000 column using an Agilent 1260/1290 Infinity II high-performance liquid chromatography.

## Data Availability

All relevant data are contained within the paper and its supporting information files.

## SUPPLEMENTAL MATERIAL

Supplemental material mbio.03202-24-s0001.pdf Supplemental tables and figures.

ASM does not own the copyrights to Supplemental Material that may be linked to, or accessed through, an article. The authors have granted ASM a non-exclusive, world-wide license to publish the Supplemental Material files. Please contact the corresponding author directly for reuse.
