# Supplementary material for: Avidity and variable domain spacing strongly influence the therapeutic potency of bispecific antibodies against Crimean-Congo hemorrhagic fever virus
Source: mBio. 2025 Apr 16;16(5):e03202-24. doi: 10.1128/mbio.03202-24 (PMC12077110; doi:10.1128/mbio.03202-24)
Supplement: Supplemental material — Supplemental tables and figures. [file mbio.03202-24-s0001.pdf]

Supplemental material for :

Avidity and variable domain spacing strongly influence the therapeutic potency of bispecific antibodies against Crimean-Congo hemorrhagic fever virus

Albert Wang,[a^] Stephanie R. Monticelli,[b,c*^] Ariel S. Wirchnianski,[a] Dafna M. Abelson,[d] Ana I. Kuehne,[b] Russell R. Bakken,[b] Marissa Middlecamp,[d] Michael Weingart,[d] Olivia Vergnolle,[e*], Zachary A. Bornholdt,[d*] Crystal L. Moyer,[d*] Jacob L. Berrigan,[a] Brandyn R. West,[d*] J. Maximilian Fels,[a*] Larry Zeitlin,[d] Andrew S. Herbert,[b§] Kartik Chandran,[a§] Chowdhury Raihan Bikash,[e*#§] Jonathan R. Lai[e#§]

DVD-Ig	Heavy chain linker	Light chain linker
DVD-121-801	ASTKGP	TVAAP
DVD-121-801^{GS}	GGGGSGGGGGSGGGG	GGSGGGGSGGGGS
DVD-121-801^{SVF}	ASTKGPSVFPLAP	TVAAPSVFIFPP
DVD-121-121	ASTKGP	TVAAP
DVD-121-121x	ASTKGP	TVAAP
DVD-121x-121	ASTKGP	TVAAP
DVD-121-F4	ASTKGP	TVAAP

Supplemental Table 1: Linkers of designed DVD-Ig molecules, related to Figure 1.

	**Virus**						
	tecVLP	CCHFV					
**Antibody**	IbAr 10200	IbAr 10200	Oman	Hoti	Turkey	Afg09	M18-China
ADI-36121	0.1082	0.0127	0.1746	0.1724	0.0829	0.0884	0.0106
ADI-37801	n/a	2.404	3.986	n/a	18.31	weak	2.799
ADI-37801^{C49}	n/a	n.d.	n.d.	n.d.	n.d.	n.d.	n.d.
121 + 801 cocktail	n.d.	0.0228	0.2287	0.2421	0.0875	0.1380	0.0181
DVD-121-801	0.0589	0.0030	0.0920	0.1015	0.0521	0.0807	0.0078
DVD-121-801^{C49}	0.0384	n.d.	n.d.	n.d.	n.d.	n.d.	n.d.
DVD-121-801^{GS}	0.0387	0.0092	0.0978	0.0683	0.0529	0.0652	0.0086
DVD-121-801^{SVF}	0.0487	0.0058	0.0591	0.0738	0.0623	0.0564	0.0079
DVD-121-121	0.0421	0.0078	0.0652	0.0630	0.0278	0.0540	0.0043
DVD-121-121x	0.1471	0.0536	0.2751	0.2259	0.1530	0.2106	0.0227
DVD-121x-121	0.6778	0.0319	0.6810	2.293	0.1402	0.7059	0.0266
DVD-121-F4	0.0630	n.d.	n.d.	n.d.	n.d.	n.d.	n.d.

Supplemental Table 2: Relative IC$_{50}$ values (nM) of parental mAbs and DVD-121-801 bsAb variants, related to Figure 2.

IC$_{50}$ values were derived from neutralization curves fitted with a four-parameter non-linear regression (**Figures S1, S2**). ADI-37801 was non-neutralizing against tecVLPs (IbAr10200), CCHFV-Hoti, and weakly neutralizing against CCHFV-Afg09. Similarly, ADI-37801^{C49} was non-neutralizing against tecVLPs. Authentic virus neutralization assays for DVD-121-F4, ADI-37801^{C49}, and DVD-121-801^{C49} were not performed.

n.d. not determined.

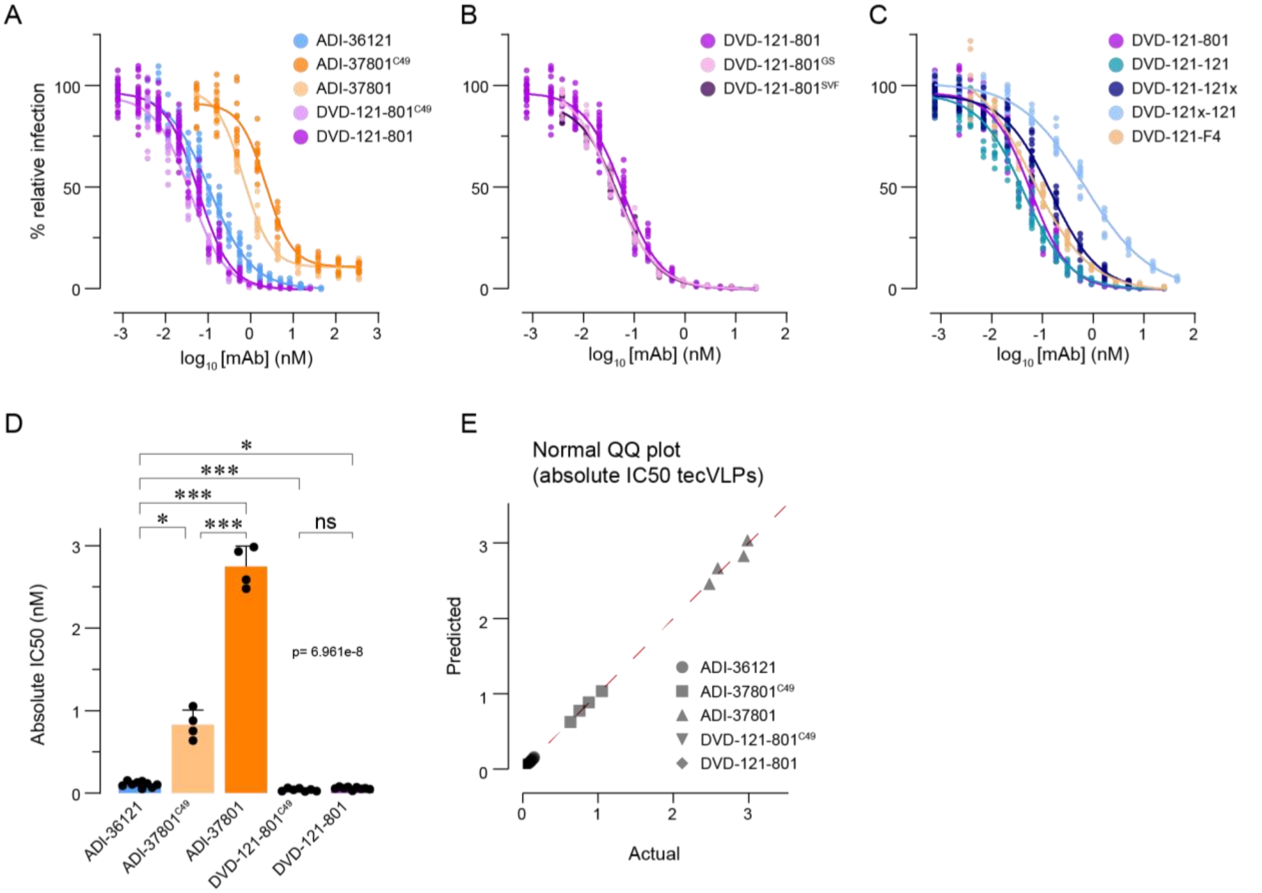

Supplemental Figure 1: TecVLP neutralization by parental mAbs and DVD-121-801 bsAb variants, related to Figure 2.

S1A-C Neutralization plots (N= 3-10, n= 9-30) of parental mAbs and DVD-Igs (**S1A**), DVD-121-801 linker variants (**S1B**), and bispecific 121 variants (**S1C**). Plots were fitted with a four-parameter non-linear regression model. Infection levels were normalized relative infected cells in the absence of antibody treatment. **S1D** Absolute IC_{50} values of the parental mAbs and DVD-Igs were compared by one-way ANOVA with Dunnett's T3 multiple comparisons test, following tests for normality (**S1E**) and homoscedasticity (not shown).

*** $p \leq 0.001$; * $p \leq 0.05$; n.s. $p > 0.05$.

1

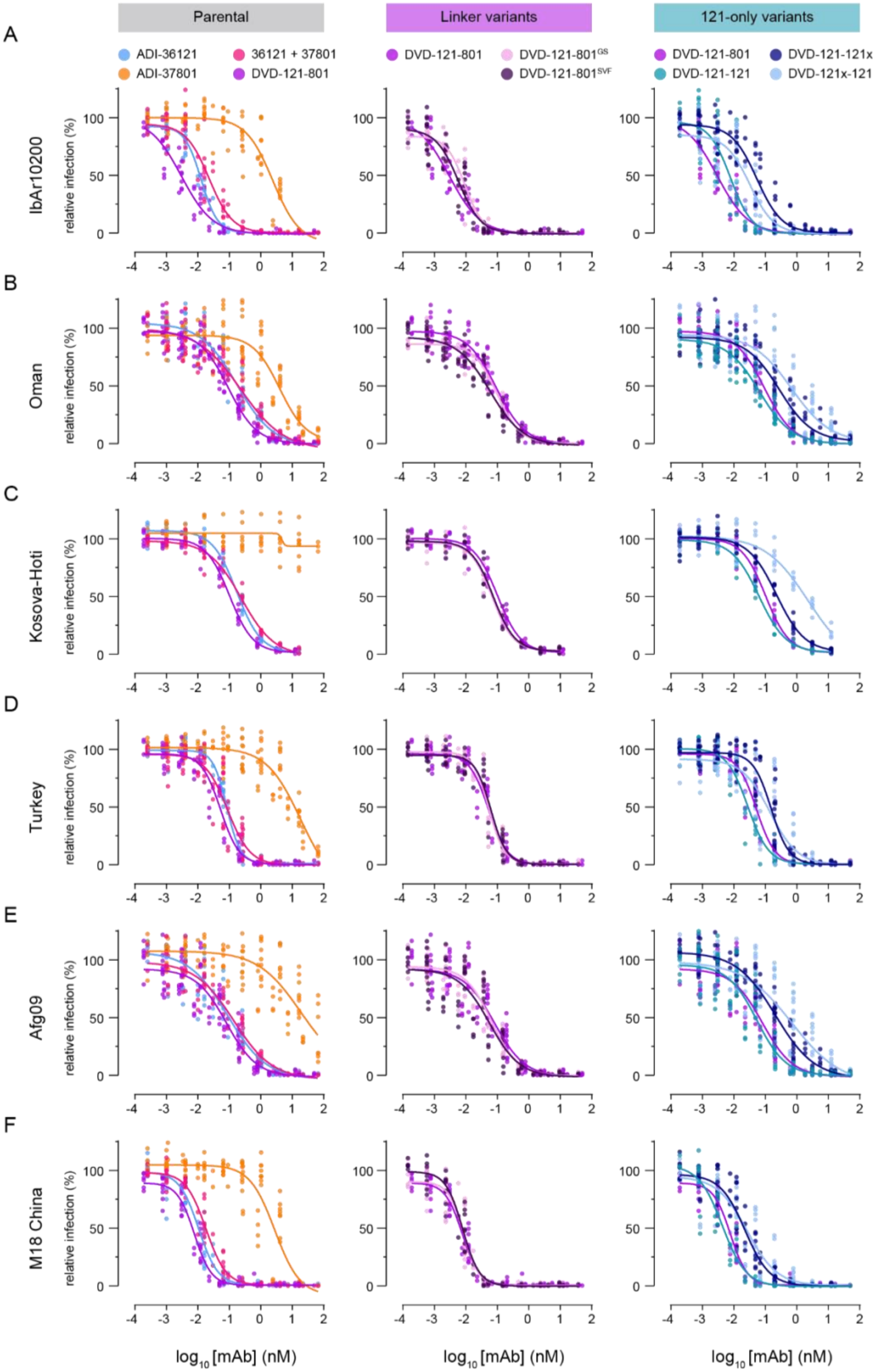

A
B
C
D
E
F
Parental
Linker variants
121-only variants
ADI-36121
ADI-37801
36121 + 37801
DVD-121-801
DVD-121-801GS
DVD-121-801SVF
DVD-121-121
DVD-121-121x
DVD-121x-121
IbAr10200
Oman
Kosova-Hoti
Turkey
Afg09
M18 China
relative infection (%)
log10 [mAb] (nM)

2

Supplemental Figure 2: Authentic CCHFV neutralization by parental mAbs and DVD-121-801 bsAb variants, related to Figure 2.

S2A-F Neutralization plots (N= 3-5, n= 8-15) of parental mAbs and DVD-Igs, DVD-121-801 linker variants, and bispecific 121 variants against CCHFV strains IbAr10200 (**S2A**), Oman (**S2B**), Hoti (**S2C**), Turkey (**S2D**), Afg09 (**S2E**), and M18 China (**S2F**) were fitted with a four-parameter non-linear regression model. Infection levels were normalized relative infected cells in the absence of antibody treatment.

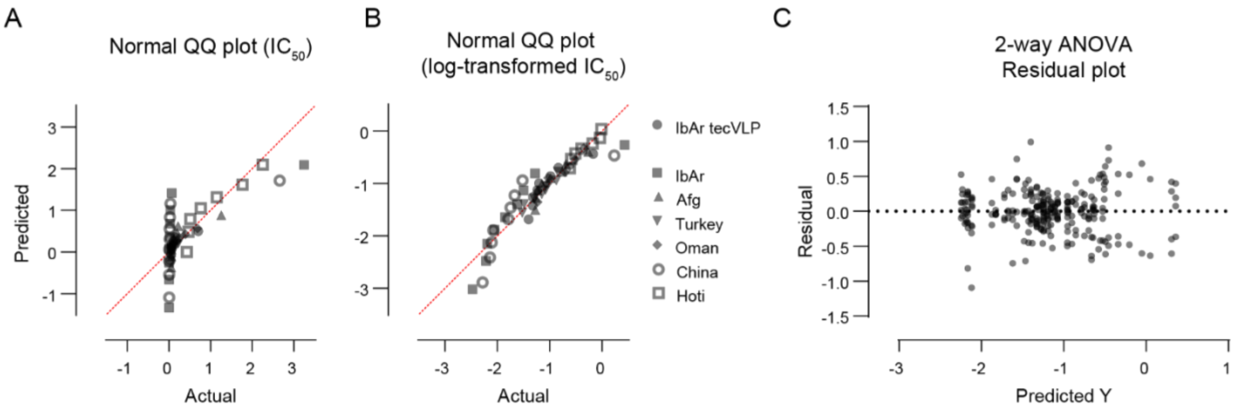

Supplemental Figure 3: 2-way ANOVA for IC_{50} comparisons, related to Figure 2

S3A-B QQ plots of IC_{50} values pre- (**S3A**) and post-log-transformation (**S3B**). **S3C** Residual plot of 2-way ANOVA comparison of IC_{50} values to evaluate model fit.

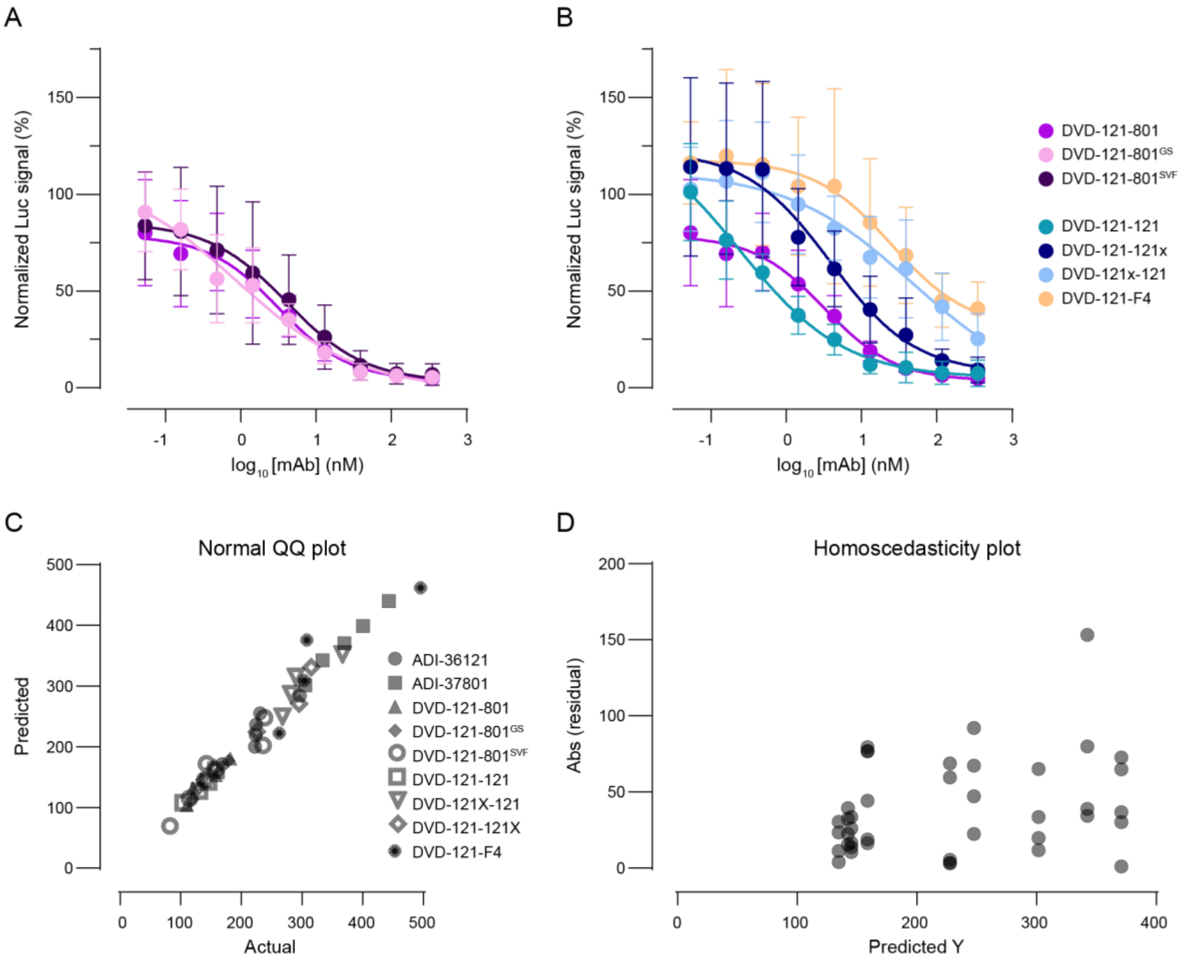

Supplemental 4: Fusion inhibition of tecVLPs (Oman) by parental mAbs and DVD-121-801 bsAb variants, related to Figure 3.

S4A-B Fusion inhibition plots (N= 4-6, n= 8-18) of linker variants (**S4A**) and 121 only variants (**S4B**) were fitted with a four-parameter non-linear regression model. The average infectivity and standard deviation for each antibody dilution is depicted. Infection levels were normalized relative to conditions with no antibody treatment. Area under the curve values were calculated and compared by one-way ANOVA with Šídák's multiple comparisons test, following tests for normality (**S4C**) and homoscedasticity (**S4D**).

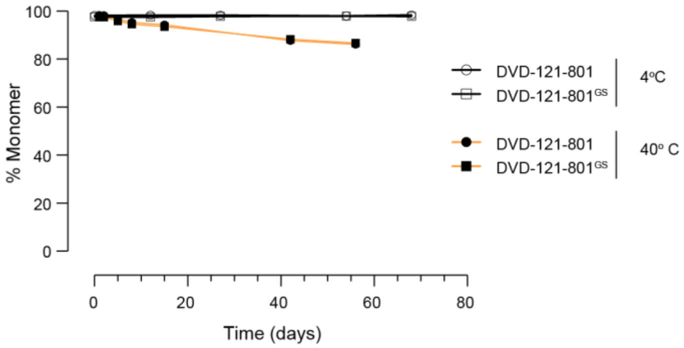

Supplemental Figure 5: Stability studies of bsAbs variants

Aliquots of the indicated bsAbs were incubated at either 0ºC or 40ºC. Samples were periodically analyzed by size-exclusion chromatography (SEC). The percentage of sample in the monomeric form is graphed.
